# Anthropometric Measurements and Lifestyle Characteristics of Individuals with Non-alcoholic Fatty Liver Disease

**DOI:** 10.7759/cureus.7016

**Published:** 2020-02-17

**Authors:** Faryal Tahir, Zainab Majid, Bushra Majid, Jawad Ahmed, Arbaz Zaman, Moeez Tariq, Fouzia Imtiaz, Syeda Anjala Tahir

**Affiliations:** 1 Internal Medicine, Dow University of Health Sciences, Karachi, PAK; 2 Genetics, Dow University of Health Sciences, Karachi, PAK; 3 Internal Medicine, Civil Hospital, Karachi, PAK

**Keywords:** non-alcoholic fatty liver disease, nafld, nash, metabolic syndrome, physical activity, insulin resistance, fatty liver, cirrhosis, body mass index, obesity

## Abstract

Background

Non-alcoholic fatty liver disease (NAFLD) is a common liver disorder caused by the deposition of lipids and fats in the hepatocytes, in individuals who consume little or no alcohol, which eventually progresses to cirrhosis and carcinoma. Apart from the known risk factors like obesity, metabolic syndrome (MS), and lack of physical activity (PA), diet also plays a major role in the development of NAFLD. A high body mass index (BMI) and waist circumference (WC) have positive associations with NAFLD. The aim of this study was to find the prevalence of risk factors of hepatic steatosis in NAFLD population and to raise public awareness about the condition.

Method

We conducted a cross-sectional study from October to December 2019 with a sample size of 98 subjects determined by using a confidence interval (CI) of 99.9%. Patients presenting to Essa Laboratory, Karachi for abdominal ultrasound (US) were scanned for fatty changes in the liver, after obtaining consent, and were then assessed for risk factors by administering a 20-item questionnaire along with registering their BMI and WC measurement. The collected data was analyzed using the Statistical Package for Social Sciences (SPSS), version 22 (IBM, Armonk, NY). The independent sample t-test was applied for the exploration of variables and a p-value <0.05 was considered significant.

Result

Our study included 96 participants, of which 49 (51%) were male and 47 (49%) female. Mean BMI in females was slightly greater (30.58) than in males (27.98), whereas WC (in inches) was almost equal in males (40.796) and females (40.383). Among the people that had any comorbidities (n = 60, 62.5%), hypertension (HTN) was the most common one (n = 37, 38.5%) followed by diabetes mellitus (DM) type 2 (n = 26, 27.1%). A significant majority (n = 63, 65.5%) never consumed any fruits or vegetables in their meal nor did they perform any sort of physical exercise (n = 46, 47.9%).

Conclusion

Obesity (high BMI), lack of PA, lower consumption of fruits and vegetables along with a carbohydrate- and fat-rich diet play a vital role in the development of hepatic steatosis. Moreover, HTN and DM, as components of MS, exhibit a significant association with NAFLD. Screening and counseling sessions should be considered for individuals with these anthropometric measurements and lifestyle characteristics.

## Introduction

Non-alcoholic fatty liver disease (NAFLD) is one of the most common liver disorders worldwide. It consists of a variety of liver disorders characterized by fatty changes (hepatic steatosis) in more than 5% of hepatocytes in the absence of excessive alcohol intake. NAFLD has a variable presentation, ranging from being a silent metabolic disorder to a symptomatic non-alcoholic steatohepatitis (NASH). It eventually progresses to cirrhosis and, in serious cases, hepatocellular carcinoma (HCC) [1]. The prevalence of NAFLD is lower in Asia (15-20%) as compared to the West (20-30%) [2].

Recent available data suggest that NAFLD develops due to the deposition of lipids and fats in the liver hepatocytes. Many risk factors play a vital role in the accumulation of this fat, namely the type and contents of the diet, obesity, metabolic syndrome (MS), and lack of physical activity (PA). Diet plays a significant role in the development of NAFLD. Increased intake of carbohydrates, animal proteins, and sugar are linked to the onset of NAFLD [3-5]. Excess carbohydrate use can lead to insulin resistance (IR), which itself is also a contributing factor for NAFLD [3]. MS is a cluster of metabolic derangements characterized by obesity, raised blood pressure (BP), raised blood glucose levels, IR, and dyslipidemia. MS and obesity are also found to be closely linked to NAFLD [6-8]. In recent decades, as the incidence of MS has peaked, NAFLD has also been on the rise. Obesity, another major risk factor for NAFLD, disturbs the oxidative pathways in adipose tissues and promotes IR. This oxidative stress creates unfavorable conditions for the liver and worsens NAFLD [6]. An increase in the body mass index (BMI) and waist circumference (WC) are positively associated with the progression of hepatic steatosis. Both these factors show a significant association with MS and IR as well [5].

Lack of PA has been associated with deterioration and progression of NAFLD [6,9,10]. PA promotes better health and has been proven to be beneficial in reducing IR and improving lipid levels in the blood [6].

A majority of NAFLD patients are obese or overweight and are in their 4th-6th decade of life. These patients are usually asymptomatic clinically, but may also present with hepatosplenomegaly, fatigue, dyspepsia, and discomfort in the right upper quadrant (RUQ) where the liver is located. Cirrhosis may develop, and patients may present with jaundice, ascites, or variceal bleed. Patients may also present with a clinical picture of MS and IR as seen in diabetes mellitus (DM) [1,6,7]

The diagnosis of NAFLD can be made on the basis of clinical features aided by laboratory investigations and imaging modalities. Levels of alanine aminotransferase (ALT) and aspartate aminotransferase (AST) may be raised but AST/ALT ratio is <1 in NAFLD [1]. Lipid profile, blood glucose levels, and calculation of BMI may aid in diagnosing coexisting MS or DM. Abdominal ultrasound (US) is the first line and the most commonly used imaging modality for NAFLD. Unfortunately, the results of the US are unreliable in obese patients. CT scan and MRI are the other non-invasive imaging modalities. Fibroscan (transient elastography), magnetic resonance elastography (MRE), acoustic radiation force impulse elastography (ARFIE) are used to assess stiffness and fibrosis in the liver during later stages of NAFLD. However, biopsy still remains the gold standard for the investigation for NAFLD [1,6,11,12].

The primary aim of this study was to assess the prevalence of risk factors of hepatic steatosis including BMI, WC, dietary habits, PA, and comorbidities among the NAFLD population. The secondary objective was to increase awareness among public health practitioners regarding the lifestyle characteristics of people with NAFLD.

## Materials and methods

We conducted a cross-sectional study from October 2019 to December 2019 in order to evaluate the association of NAFLD in the general population of Karachi with their diet, lifestyle, and comorbidities. The sample size for our survey was calculated using OpenEpi.com with a confidence level of 99.9%, leading us to opt for 98 participants. In order to facilitate the screening of participants in our study, we collaborated with Essa Laboratory, Karachi to access their liver US system, which was our screening modality. We selected the US on account of it being cheap, non-invasive, and readily available.

We included participants who presented to the Essa laboratory for the abdominal US for various causes. A liver US was performed at the same time on the participants to assess the presence of hepatic steatosis after getting their written and verbal consent. Individuals with increased echogenicity on the US (suggestive of fatty changes), regardless of the level, were considered as subjects of our study and were selected for further evaluation via our questionnaire and the evaluation of BMI and WC. Exclusion criteria included people who were less than 18 years of age and those who were unable to communicate due to language or physical/health barriers. People with other hepatic pathologies were also excluded.

Survey design

A self-administered 20-item questionnaire was formulated after going through relevant published articles. Our survey tool was divided into five parts, the first being a demographic one which included age, gender, occupation, and socioeconomic status and WC (inches). The weight (Kg) and height (m) were also measured for the evaluation of BMI. The various occupations were classified using collar colors, the salaried professionals being referred to as white-collar, manual laborers as blue-collar, government workers as red, and those in the service industry as pink-collar [13]. The second part assessed dietary habits including participants' dinner details, preferable choice of food, the proportion of fruits and vegetables, and consumption of junk foods (soft drinks, bakery, and canned items). The next two parts focused on PA and stress levels of our participants. The last section analyzed the comorbidities, significant family history, and a brief drug history.

Prior to our data collection, a pilot study involving three volunteers was carried out in order to mold our survey tool into a more relevant and easier one. We approached patients who approached the Essa Laboratory for abdominal US and, after thoroughly guiding them about the evaluation process, consent was taken. Each participant who had undergone the liver US had their weight, height, and WC analyzed by the trained members of our research team. Lastly, the subjects were asked to fill in the details of our questionnaire.

Statistical analysis

Out of the 98 participants, two were excluded due to incomplete responses. The collected data was analyzed using the Statistical Package for Social Sciences (SPSS), version 22 (IBM, Armonk, NY). For the evaluation of categorical variables, frequencies and percentages were used whereas for continuous variables, the mean and standard deviation (SD) were reported. The independent sample t-test was applied for the exploration of variables and a p-value <0.05 was deemed significant.

## Results

Socio-demographic characteristics

Our study included 96 participants of which 49 (51%) were male and 47 (49%) female. Of the participants, 28 (29%) were aged between 41 and 50 years, and 24 were between 31 and 40 years (25%). Only 9 (9.4%) were greater than 60 years in age. Significantly, 81.3% (n = 78) belonged to the middle class, while the remaining were from low- and high-classes. Almost two-fifth (39.6%, n=38) of the female participants were housewives. Of note, 25% (n = 24) of the participants belonged to the blue-collar, 12.5% (n=12) were categorized as white-collar, while only 5.2% (n=5) of them were unemployed (Table 1).

**Table 1:** Demographics of the participants (n = 96)

Variables	Number of participants	Percentage (%)
Age (years)		
18-20	4	4.2
21-30	18	18.8
31-40	24	25.0
41-50	28	29.2
51-60	13	13.5
>60	9	9.4
Gender		
Male	49	51
Female	47	49
Occupation		
Housewife	38	39.6
Blue collar	24	25
White collar	12	12.5
Red collar	4	4.2
Pink collar	4	4.2
Retired	3	3.1
Unemployed	5	5.2
Students	6	6.25
Status		
Low class	13	13.5
Middle class	78	81.3
High class	5	5.2

Body mass index and waist circumference

BMI of each participant was calculated by measuring their height in meters (m) and weight in kilograms (Kg). In addition, WC was measured in inches. Mean BMI in our study participants was found to be 29.26, while mean WC was calculated to be 40.59. Mean BMI in females was slightly greater (30.58) than in males (27.98) whereas the WC was almost equal in males (40.796) and females (40.38). Mean BMI (34.64, n = 5) and WC (44.70, n = 5) were seen highest among the high-class group in contrast to the middle and low class whose mean BMI were 28.94 (n = 78) and 29.05 (n = 13) and mean WC were 40.28 (n = 78) and 40.92 (n = 13), respectively. There was also a significant association between mean BMI and gender (p: 0.006) (Table 2).

**Table 2:** Mean BMI and mean WC of participants with respect to gender and social status (n = 96) BMI: body mass index; WC: waist circumference

Variables	Number of participants	Mean BMI (Kg/m^2^)	Mean WC (Inches)	P-value
Gender				
Male	49	27.98	40.796	0.006 (for mean BMI)
Female	47	30.58	40.383	
Status				
Low class	13	29.05	40.923	
Middle class	78	28.94	40.276	
High class	5	34.64	44.700	

Assessment of dietary habits, lifestyle, and comorbidities

The lifestyle of our study participants was assessed via a structured questionnaire, which examined their dietary and PA habits along with any comorbidities in themselves and/or in their family as well as a brief drug history. Almost half of the study participants (n = 52, 54.2%) preferred to eat meat with either rice or roti (bread) at dinner. Those who dined out mostly preferred to have barbeque (n = 23, 24%). A significant majority (n = 63, 65.5%) never consumed any fruits or vegetables in their meal nor did they perform any sort of physical exercise (n = 46, 47.9%). The majority preferred using stairs (n = 59, 61.5%) as the medium of ascend while one-fourth (n = 28, 29.2%) claimed that their occupation did not involve any PA. Of the 40% (n = 39) of the participants who had a prior test of cholesterol, approximately two-fifth had abnormal levels (n = 15, 15.6%). Among the people who had any comorbidities (n = 60, 62.5%), hypertension (HTN) was the most common (n = 37, 38.5%). The same was evident for any comorbidities among their family members (n = 51, 53.1%). Subsequently, anti-hypertensives were the most commonly taken drugs by our study participants (n = 32, 33.3%). The responses for the assessment of risk factors are summarized in Table 3.

**Table 3:** Assessment of dietary habits, lifestyle, comorbidities, and stress levels among participants (n = 96) PA: physical activity; DM: diabetes mellitus; HTN: hypertension; CVD: cardiovascular disease: CKD: chronic kidney disease

Questions	Options, n (%)							
Q1. Meals consumed in a day	1	2	3	4	>4			
A1.	2 (2.1)	24 (25.0)	62 (64.6)	5 (5.2)	3 (3.1)			
Q2. Preference to eat in dinner	Only meat	Meat with rice or roti (bread)	Only rice	Others				
A2.	8 (8.3)	52 (54.2)	22 (22.9)	14 (14.6)				
Q3. Time taken to complete dinner	<10 mins	Between 10-15 mins	Between 15-25 mins					
A3.	21 (21.9)	58 (60.4)	17 (17.7)					
Q4. Time of dinner	Before 9 pm	After 9 pm	At midnight	No exact time				
A4.	25 (26.0)	45 (46.9)	14 (14.6)	12 (12.5)				
Q5. Frequency of eating out	Never	Once in a month	Once in every 15 days	Once in a week	Daily	More than once in a week		
A5.	19 (19.8)	31 (32.3)	16 (16.7)	18 (18.8)	11 (11.5)	1 (1)		
Q6. Preference of meal when dining out	Not applicable	Chinese	Grilled	Barbeque	Fast food	Continental	Multiple preferences	Desi (Pakistani)
A6.	19 (19.8)	2 (2.1)	2 (2.1)	23 (24.0)	19 (19.8)	8 (8.3)	17 (17.7)	6 (6.3)
Q7. Frequency of eating fruits and vegetables	Never	Once in a month	Once in every 15 days	Once in a week	Daily			
A7.	63 (65.5)	21 (21.9)	4 (4.2)	5 (5.2)	3 (3.1)			
Q8. Consumption of canned foods	Never	Once in a month	Once in every 15 days	Once in a week	Daily			
A8.	63 (65.6)	21 (21.9)	4 (4.2)	5 (5.2)	3 (3.1)			
Q9. Consumption of bakery items	Never	Once in a month	Once in every 15 days	Once in a week	Daily			
A9.	16 (16.7)	13 (13.5)	9 (9.4)	24 (25.0)	34 (35.4)			
Q10. Consumption of cold/sugary/fizzy drinks	Never	Once in a month	Once in every 15 days	Once in a week	Daily	More than once in a week		
A10.	20 (20.8)	20 (20.8)	20 (20.8)	28 (29.2)	7 (7.3)	1 (1)		
Q11. Frequency of workout	Never	Once in a month	Once in every 15 days	Once in a week	Daily			
A11.	46 (47.9)	19 (19.8)	5 (5.2)	13 (13.5)	13 (13.5)			
Q12. Preference for medium of ascend	No Preference	Stairs	Escalator	Lift				
A12.	13 (13.5)	59 (61.5)	3 (3.1)	21 (21.9)				
Q13. Involvement of PA at occupation	Never	Sometimes	Always					
A13.	28 (29.2)	34 (35.4)	34 (35.4)					
Q14. Frequency of getting irritated	Never	Sometimes	Frequently	Always				
A14.	22 (22.9)	40 (41.7)	19 (19.8)	15 (15.6)				
Q15. Sleep with a relaxed mind	No	Yes	Sometimes					
A15.	18 (18.8)	59 (61.5)	19 (19.8)					
Q16. Number of causes of stress	None	A few	Too many					
A16.	32 (33.3)	47 (49.0)	17 (17.7)					
Q17. Any comorbidities	No	Yes						
A17a.	36 (37.5)	60 (62.5)						
17b. DM Type 2	70 (72.9)	26 (27.1)						
17c. HTN	59 (61.5)	37 (38.5)						
17d. Hepatitis	91 (94.8)	5 (5.2)						
17e. CVD	88 (91.7)	8 (8.3)						
17f. CKD	89 (92.7)	7 (7.3)						
17g. Others	86 (89.6)	10 (10.4)						
Q18a. Cholesterol ever tested	No	Yes						
A18a.	57 (59.4)	39 (40.6)						
18b. If yes, what were the levels?	Not applicable	Normal	Abnormal	Not Known				
A18b.	57 (59.4)	23 (24.0)	15 (15.6)	1 (1)				
Q19a. Comorbidities in the family	None	Yes						
Ans19a.	18 (18.8)	78 (81.3)						
19b. DM Type 2	52 (54.2)	44 (45.8)						
19c. HTN	45 (46.9)	51 (53.1)						
19d. CVD	65 (67.7)	31 (32.3)						
19e. Hyperlipidemia	73 (76)	23 (24)						
Q20a. Medications taken regularly	No	Yes						
A20a.	52 (54.2)	44 (45.8)						
20b. Antidepressants	92 (95.8)	4 (4.2)						
20c. Antiepileptic	94 (97.9)	2 (2.1)						
20d. Diabetes medicines	73 (76)	23 (24)						
20e. Steroids	94 (97.9)	2 (2.1)						
20f. Anti-hypertensives	64 (66.7)	32 (33.3)						
20g. Others	93 (96.9)	3 (3.1)						

Comorbidities among the participants (n = 60, 62.5%)

**Figure 1:**
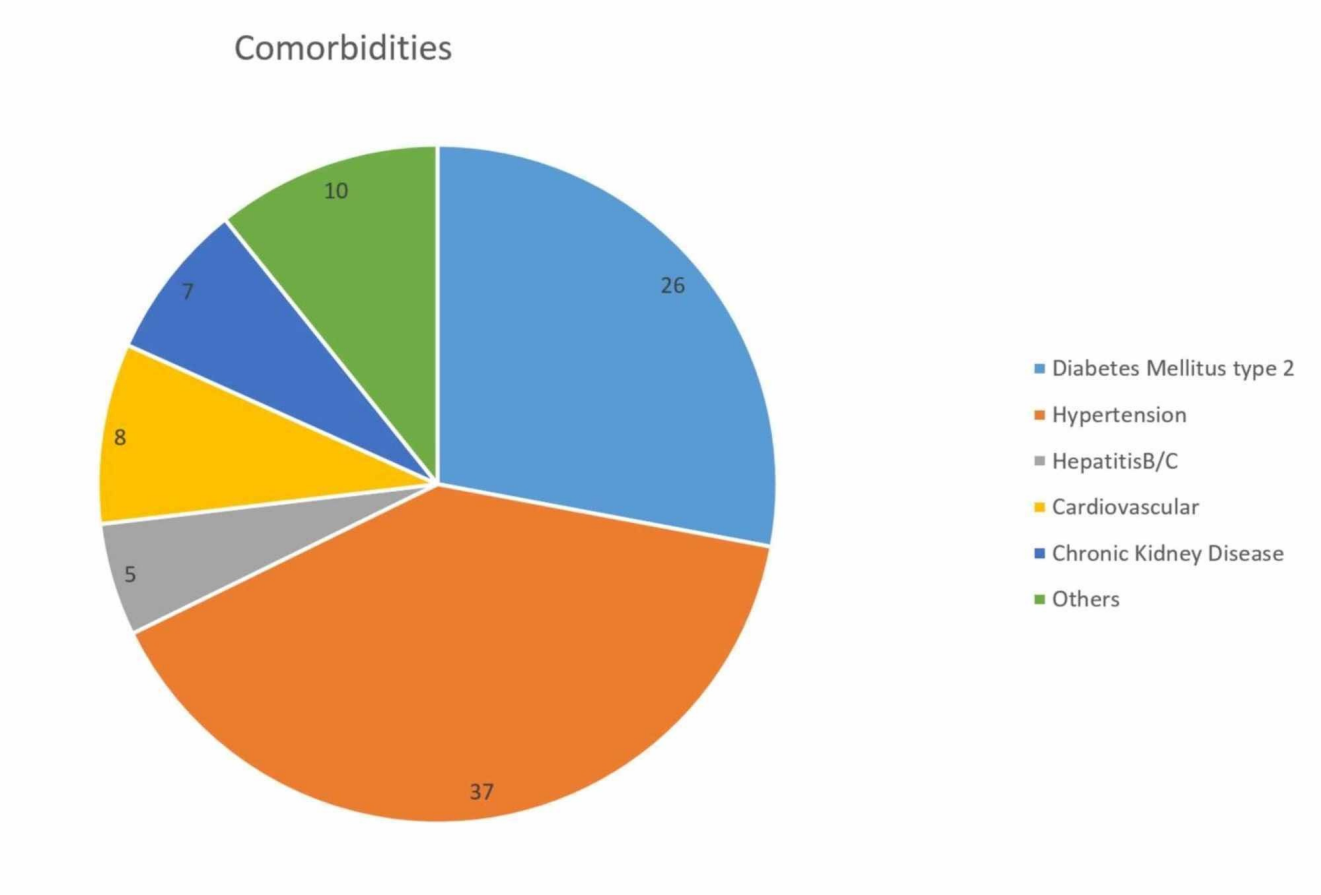
Major comorbidities among participants (n = 60)

A total of 62.5% (n = 60) participants had comorbidities, either single or multiple. HTN was found to be the most prevalent comorbidity (n = 37, 38.5%) followed by DM type 2 (n = 26, 27.1%) (Figure 1).

**Figure 2:**
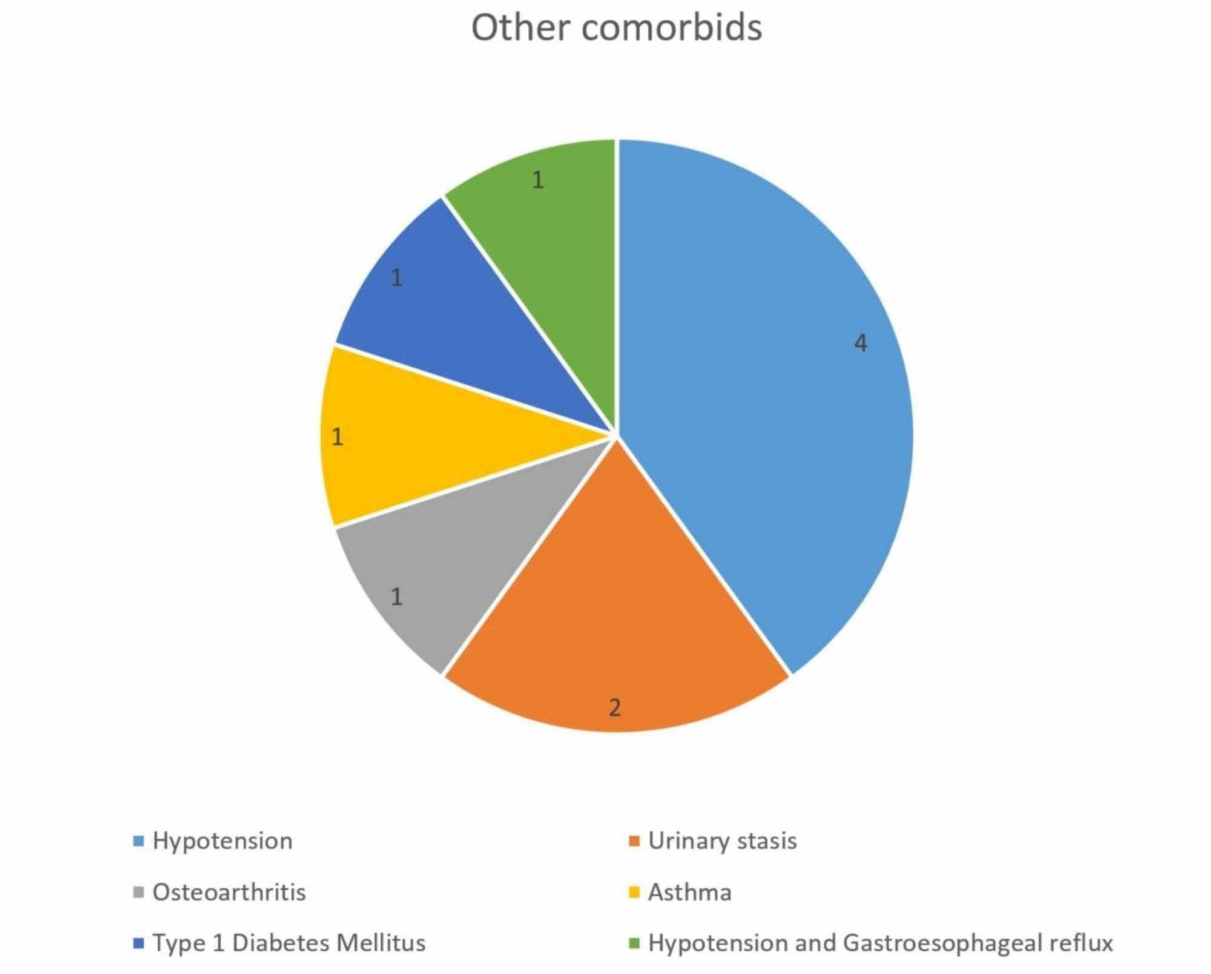
Other comorbidities among participants (n = 10)

Ten (10.4%) of the participants had comorbidities other than DM type 2, HTN, hepatitis, cardiovascular disease (CVD) and chronic kidney disease (CKD). These are enlisted with respect to their frequencies in Figure 2.

## Discussion

With a rising incidence of non-communicable diseases around the world, NAFLD has become a new challenge for public health. The prevalence of NAFLD across the world has been reported to be 10-30%, whereas it is estimated to be 15-20% in Asia [2,11]. With a peak in incidences of obesity and diabetes in Pakistan, it is reasonable to assume escalation in the frequency of NAFLD as well [14,15]. However, there is inadequate data on the prevalence of NAFLD in Pakistan, possibly because of its asymptomatic status and lack of screening strategies. According to estimated trends, NAFLD has become one of the common etiologies leading to liver failure in older adults [16]. Therefore, this study was conducted to highlight the prevalence of established risk factors for fatty liver among individuals ultrasonically diagnosed with NAFLD.

Obesity is regarded as a chronic disease defined by a BMI of more than 30 Kg/m2. NAFLD shares a strong association with obesity, with a reported prevalence as high as 80% among the obese population and only 16% in individuals with normal BMI (i.e. 18-24 Kg/m^2^) and no metabolic risk factors [15]. Hence, an increase in BMI is strongly associated with a rise in the prevalence of NAFLD [16]. It is evident from our study, as the mean BMI among the NAFLD population was found to be 29.25 Kg/m^2^. The same results were reported by Rocha et al., who established the highest prevalence rates of BMI and WC among individuals with NAFLD and concluded that WC might be the best predictor of NAFLD [5]. Although hepatic steatosis has been associated with BMI, a higher correlation between the occurrence and progression of NAFLD and visceral adiposity (measured as WC) has been appreciated, in part due to visceral adipose tissue being more active (lipolitically) than subcutaneous fat [1]. Subsequently, a direct release of free fatty acids (FFAs) into the circulation is considered a significant mediator of IR. Secondary to IR, obesity stimulates various inflammatory pathways in the adipose tissue. Consequently, oxidative stress plays a vital role in the development and progression of NAFLD [6]. Therefore, individuals with central obesity are considered more prone to develop NAFLD. A recent meta-analysis has shown a substantial rise in the incidence of NAFLD with increasing BMI and WC measurements [17].

Solga et al. suggested that total calorie intake and macro- and micronutrients may play a significant role in the pathogenesis and progression of NAFLD [18]. Dietary carbohydrate consumption is a major stimulus of hepatic de novo lipogenesis (DNL). The increased intake of carbohydrate-rich diet has also been linked to IR. Therefore, evidence suggests that carbohydrate in diet plays an important role in the development of NAFLD [3]. In a study conducted among aged Caucasians, a higher intake of animal protein was found to be associated with NAFLD in overweight individuals irrespective of any well-known risk factor [4]. Almost the same results were found in our study, where half of the subjects (54.2%) preferred to eat meat with either rice or roti (bread) at dinner. Moreover, those who dined out preferred to eat barbeque (24%). Likewise, a significant majority (35.4%) in our study reported the consumption of bakery items on a daily basis. Furthermore, a vast majority (65.5%) never consumed any sort of fruits or vegetables in their meal. This is consistent with the results of a meta-analysis, where intakes of red meat, fats, and sweets were high whereas consumption of whole grains, fruits, and vegetables was found to be less among the NAFLD population [19].

Recent studies have shown a correlation between dietary sugar consumption and hepatic fat accumulation because fructose can serve as a substrate for DNL inside the liver and consequently increase triglyceride (TG) levels [16,20]. Results from a meta-analysis have shown that the intake of sweetened drinks increases susceptibility for MS, elevated TG levels, and increased visceral fat [21]. However, in our study, only one-third of the subjects (29.2%) consumed sugary drinks once a week. Similarly, excessive consumption of high fructose corn syrup, commonly added in canned foods, induces oxidative stress and increased hepatic lipogenesis [22]. In contrast, a significant majority (65.6%) in our study never consumed canned foods. Another study highlighted that patients with NAFLD can suffer from liver injury if they consume high amounts of dietary fat [23]. In epidemiological studies, both polyunsaturated and saturated fatty acids in diet have been related to the presence of NAFLD [24].

Our study also found that the frequency of PA was significantly lower among the NAFLD population where approximately half of the subjects (47.9%) never opted for a workout, which is consistent with the findings of Tayyem RF et al. [25]. According to Kenneally S et al., individuals with NAFLD spend major time in sedentary habits with less PA on a daily basis as compared to normal individuals [9]. Another research conducted among the adult population showed a conspicuous association between NAFLD prevalence and prolonged sitting time with decreased PA levels [8]. The same results were reported by Gerber et al., who concluded that the incidence of DM and NAFLD continues to escalate among individuals with a sedentary lifestyle [10].

MS, a major health dilemma, is closely associated with NAFLD [26]. It is a group of related metabolic abnormalities, including central obesity, HTN, dyslipidemia (increased serum TG level), low serum high-density lipoprotein (HDL) level and elevated serum low-density lipoprotein (LDL) level, hyperglycemia, and IR. A study conducted by Hsiao et al. concluded that severe fatty liver disease paralleled significantly with the prevalence and degree of HTN, DM, and dyslipidemia [7]. This is consistent with the findings of the current study, where HTN was found to be the most prevalent comorbidity (38.5%) followed by DM type 2 (27.1%) among the NAFLD population. Among the most common epidemics of the 21st century, NAFLD and type 2 DM often coexist and hence act as co-partners. Therefore, NAFLD incidence among the diabetic adult population reaches up to 75% of the general population [27]. Liver insulin signaling is vital for the homeostasis of energy via the regulation of glucose and lipid metabolism. But, in an insulin-resistant state, a cessation in the adjustment of this metabolism ensues hyperglycemia and dyslipidemia, which further aggravates the development of hepatic steatosis [28]. Thus, NAFLD has been regarded as the hepatic manifestation of MS [29].

A significant correlation between NAFLD and abnormal blood biomarkers of lipid metabolism such as TG, total cholesterol (TC), HDL, and LDL was concluded by Tayyem RF et al. [22]. Similar results were found by Sathiaraj et al., who stated that mean TC, low HDL, and TG were significantly higher in individuals with NAFLD [30]. However, we did not find a significant association between abnormal lipid profiles and NAFLD, possibly due to a lack of patient education regarding abnormal versus normal levels. Among the 40% of the individuals with a prior test of cholesterol, only 15.6% had abnormal levels.

There are several limitations to our study that need to be considered. Firstly, we used conventional abdominal US, which is often used as a first-line imaging modality for the evaluation of fatty liver clinically, especially for the screening of suspected NAFLD because of its low cost, wide variability, and non-invasiveness [12]. Secondly, the data was collected from one laboratory only, via convenience sampling. Thirdly, the exact criteria for the assessment of comorbidities were not used.

## Conclusions

NAFLD is one of the leading causes of morbidity worldwide. It may be clinically silent or may present as signs of liver cirrhosis such as ascites, hepatosplenomegaly, and abdominal discomfort in later stages. Obesity (high BMI), lack of PA, and an unhealthy diet play an important role in the development of NAFLD. A poor diet containing excess carbohydrates and increased intake of sugars and animal protein contributes to the accumulation of fat in liver cells. MS and IR have been closely linked to NAFLD. Poor control of all these risk factors can cause the progression of NAFLD to NASH and, eventually, HCC.

Starting a healthy diet with adequate proportions of carbohydrates, sugars, fats, and proteins along with engagement in PA can help control and even reverse fat accumulation in the hepatocytes. Lowering BMI and maintaining good control of blood sugar levels (DM) and BP (HTN) can prevent the development of NAFLD. Obese and hypertensive individuals, diabetic patients, and individuals with unhealthy lifestyles should be counseled regarding the implications of unhealthy and sedentary lifestyles and, particularly, the consequent adverse effects on the liver, which can lead to NASH and, in severe and untreated cases, HCC.
